# Correction to: Evolution of surgical approach to rectal cancer resection: A multinational registry assessment

**DOI:** 10.1007/s00384-024-04599-7

**Published:** 2024-02-08

**Authors:** Julie M. L. Sijmons, Jan Willem T. Dekker, Jurriaan B. Tuynman, Helen M. Mohan, Philip Smart, Alexander G. Heriot, Kate Walker, Angela Kuryba, Peter Matthiessen, Pieter J. Tanis, International Colorectal Cancer Registry Collaboration (ICORC) Collaborators, Tarik Sammour, Hidde Kroon, Sze-Lin Peng, Neal Rawson, Shoni Philpot, Ian Hayes, Lene Hjerrild Iversen, Jon Kroll Bjerregaard, Camilla Qvortrup, Ismail Gögenür, Richard Spence, Rob Tollenaar, Roel Hompes, Federico Ghignone, Helen Blake, Nicola Fearnhead, Jan van der Meulen, Mike Braun, Arne Wibe, Janet Graham, Graham Mackay, David Morrison, Ingvar Syk, Clifford Ko, Nicolas Avellaneda

**Affiliations:** 1 Dutch ColoRectal Audit (DCRA), Leiden, Netherlands; 2 Bowel Cancer Outcomes Registry (BCOR), Melbourne, Australia; 3 National Bowel Cancer Audit (NBOCA), Leeds, UK; 4 Swedish ColoRectal Cancer Registry (SCRCR), Umea, Sweden

**Correction to: International Journal of Colorectal Disease (2024) 39:15** 10.1007/s00384-023-04578-4.

In this article the International Colorectal Cancer Registry Collaboration (ICORC) Collaborators member 'Tarik Sammour' was incorrectly written as 'Tarik Samour'.

The original article has been corrected.

